# Targeting Myeloid Cells in Head and Neck Squamous Cell Carcinoma: A Kinase Inhibitor Library Screening Approach

**DOI:** 10.3390/ijms252212277

**Published:** 2024-11-15

**Authors:** Mohamed Y. Zaky, Jessy John, Monika Vashisht, Priya Singh, Mohammad A. I. Al-Hatamleh, Karen Siddoway, Zhangguo Chen, Jing H. Wang

**Affiliations:** 1 UPMC Hillman Cancer Center, Division of Malignant Hematology and Medical Oncology, Department of Medicine, University of Pittsburgh School of Medicine, Pittsburgh, PA 15224, USA; myk29@pitt.edu (M.Y.Z.); jej94@pitt.edu (J.J.); mov10@pitt.edu (M.V.); prs243@pitt.edu (P.S.); maa879@pitt.edu (M.A.I.A.-H.); kas894@pitt.edu (K.S.); zhc90@pitt.edu (Z.C.); 2 Department of Immunology, University of Pittsburgh School of Medicine, Pittsburgh, PA 15224, USA

**Keywords:** myeloid cells, head and neck squamous cell carcinoma, tumor-associated macrophages, tumor microenvironment, therapeutic targets, kinase inhibitors

## Abstract

Head and neck squamous cell carcinoma (HNSCC) is highly enriched with tumor-infiltrating myeloid cells, including tumor-associated macrophages (TAMs) and myeloid-derived suppressor cells (MDSCs). However, effective therapeutic agents targeting tumor-associated myeloid cells in HNSCC are currently lacking. Here, we employed a unique co-culture system to investigate how HNSCC cells affect tumor-associated myeloid cells. We found that the presence of cancer cells significantly enhances myeloid cell proliferation and promotes TAM differentiation. To identify potential therapeutic agents, we screened a custom library of 70 kinase inhibitors to assess their effects on distinct subsets of tumor-associated myeloid cells. We discovered specific inhibitors that differentially suppressed the populations of TAMs, monocytic MDSCs (M-MDSCs), or polymorphonuclear MDSCs (PMN-MDSCs), suggesting that inhibiting different targets could reduce distinct subsets of tumor-associated myeloid cells. Conversely, some inhibitors were found to increase the population of CD11b^+^Ly6G^−^Ly6C^−^ myeloid cells. Among the promising inhibitors tested, vatalanib, a VEGF-R inhibitor, demonstrated significant in vivo efficacy at inhibiting tumor growth and reducing tumor-associated myeloid cells, thereby underscoring its potential as a therapeutic agent. Our findings highlight specific kinase inhibitors with differential modulatory effects on HNSCC-associated myeloid subsets and caution the application of some as anti-cancer drugs. This experimental system may provide a robust platform for identifying new agents targeting tumor-associated myeloid cells in HNSCC and beyond, and for elucidating mechanistic insights into tumor-myeloid cell interaction.

## 1. Introduction

Head and neck squamous cell carcinoma (HNSCC) is a major clinical challenge because of its aggressive characteristics and the complex nature of its tumor microenvironment (TME). HNSCC can be attributed to various factors, including human papillomavirus (HPV) infection in HPV-positive cases and exposure to carcinogens such as tobacco in HPV-negative cases. HNSCC is highly enriched with myeloid cells, including tumor-associated macrophages (TAMs) and myeloid-derived suppressor cells (MDSCs), which are essential in driving tumor growth, avoiding immune detection, and resisting therapy [1,2,3,4]. However, there is limited understanding of how tumor-intrinsic factors affect the differentiation or expansion of various myeloid subsets within HNSCCs.

TAMs and MDSCs are crucial in establishing an immunosuppressive TME [3,5]. TAMs originate from tissue-resident macrophages or bone marrow (BM)-derived myeloid precursors and can shift to a pro-tumorigenic M2-like state that aids in tumor growth, metastasis, and immune suppression [6]. MDSCs, a diverse group of myeloid cells, accumulate in the TME and contribute to immune suppression, further undermining effective anti-tumor immunity [7]. These intricate interactions between myeloid cells and HNSCC cells significantly complicate the management and treatment of HNSCC [8,9].

Despite their crucial role in HNSCC progression, effective therapeutic strategies targeting tumor-infiltrating myeloid cells remain limited [9,10,11]. Conventional therapies have not sufficiently addressed the need for selective targeting of myeloid cell subsets, and there is a pressing need for novel therapeutic approaches that can modulate these cells effectively without causing systemic toxicity [12]. Recent advancements in understanding the molecular mechanisms governing myeloid cell behavior have highlighted several potential therapeutic targets, including specific kinases involved in myeloid cell differentiation and function [13].

In this study, we employed a novel co-culture system to explore how HNSCC cells influence the proliferation and differentiation of tumor-associated myeloid cells. We focused on evaluating a custom library of 70 kinase inhibitors to identify compounds that can selectively affect distinct subsets of myeloid cells. Our findings revealed that specific kinase inhibitors modulate the populations of TAMs, monocytic MDSCs (M-MDSCs), and polymorphonuclear MDSCs (PMN-MDSCs), offering insights into potential therapeutic strategies for targeting distinct subsets.

## 2. Results

### 2.1. Tumor Cells Promote Myeloid Cell Proliferation

**Figure 1:**
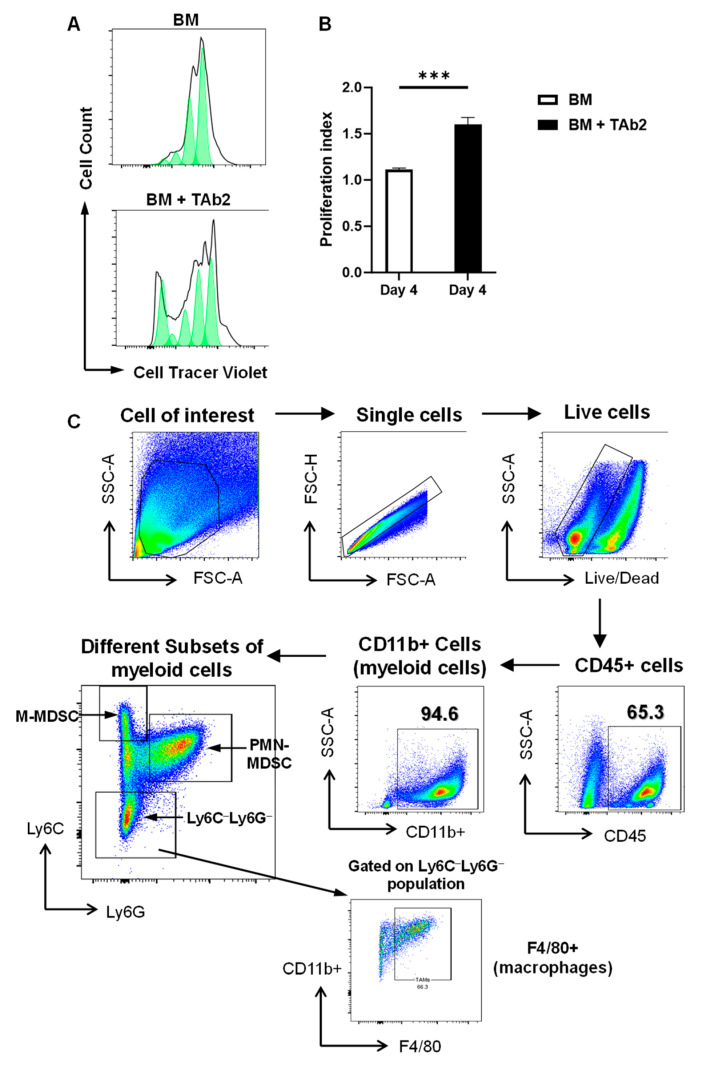
Tumor cells promote myeloid cell proliferation. (**A**) Proliferation assay. Bone marrow (BM) cells were labeled with CellTrace™ Violet and cultured for 4 days either alone or with TAb2 tumor cells. Cells were harvested and analyzed by flow cytometry on day 4. The lower intensity of CellTrace™ Violet staining indicates more cellular proliferation (bottom panel: BM+TAb2) compared to control (top panel: BM). Representative results are shown from three independent experiments performed in triplicate. (**B**) Quantification of proliferation index on day 4. Bar graphs: means ± s.e.m, White: BM cells; Black: BM+TAb2 tumor cells. Statistical significance was calculated using a student’s *t*-test, *** *p* < 0.001. Results are shown from three independent experiments. (**C**) Gating strategy for subsets of myeloid cells. After gating on the CD45^+^ population, the CD11b^+^ population was gated for myeloid cells. Within the CD11b^+^ population, M-MDSCs (Ly6C^high^Ly6G^−^), PMN-MDSCs (Ly6C^low^Ly6G^+^), and double-negative (Ly6C^−^Ly6G^−^) populations were shown. After gating on the Ly6C^−^Ly6G^−^ population, TAMs were shown (F4/80^+^CD11b^+^).

To test how tumor cells affect the proliferation and differentiation of myeloid cells, we employed a co-culture system established previously by our group [14] using BM cells that contain myeloid precursors and are co-cultured with TAb2 tumor cells. TAb2 tumor cells were derived from squamous cell carcinoma (SCC) that harbored TP53 deletion and PIK3CA hyperactivation [14]. We labeled the BM cells with CellTrace Violet, which allows the evaluation of cell proliferation because populations with less Violet staining intensity indicate more cell divisions. Our data showed that the presence of tumor cells substantially promoted the proliferation of BM cells as more division was identified by the dilution of CellTrace Violet in proliferating cells (Figure 1A). The proliferation index was significantly higher in the group of BM plus TAb2 tumor cells than in BM alone (Figure 1B).

We further examined the BM cells in the co-culture and showed that the vast majority of them were CD11b^+^ myeloid cells constituting >90% of the CD45^+^ population (a marker for hematopoietic cells) (Figure 1C). Among CD11b^+^ myeloid cells, more subsets were identified including M-MDSC (CD11b^+^Ly6G^−^Ly6C^high^) and PMN-MDSC (CD11b^+^Ly6G^+^Ly6C^low^) as well as the Ly6C^−^Ly6G^−^ population (Figure 1C, Supplemental Figure S1). When we gated on the CD11b^+^Ly6C^−^Ly6G^−^ population, most of this population consisted of F4/80^+^ macrophages, namely TAMs (Figure 1C). Thus, we conclude that the presence of tumor cells significantly promotes myeloid cell proliferation.

### 2.2. Inhibitors That Reduced Distinct Subsets of Tumor-Associated Myeloid Cells

**Figure 2:**
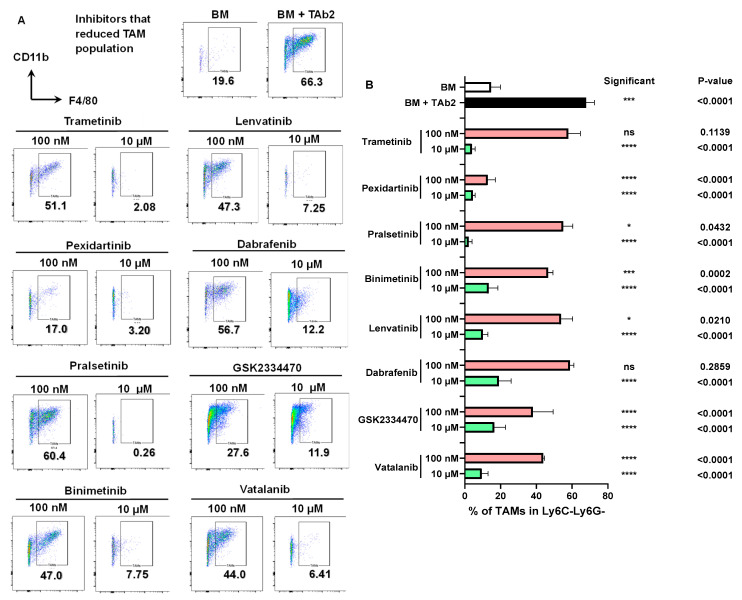
Inhibitors that reduced TAM Population. (**A**), Representative flow plots of CD11b^+^F4/80^+^ TAMs in co-culture assays. BM and BM+TAb2 serve as the negative and positive control, respectively. Indicated kinase inhibitors (100 nm or 10 μM) were added into co-culture. Cells were analyzed 4 days after culture. TAMs: CD11b^+^Ly6C^−^Ly6G^−^F4/80^+^. (**B**) Percentage of TAMs in the Ly6C^−^Ly6G^−^ population. *p*-values of comparisons between BM (white bar) vs. BM+TAb2 (black bar) or between a given group (red or green bar) vs. BM+TAb2 group (black bar, positive control) were determined using one-way ANOVA with Tukey’s multiple comparison test. Statistical significance was defined as * *p* < 0.05, *** *p* < 0.001, **** *p* < 0.0001, ns: non-significant. Results are from three independent experiments performed in triplicate.

We assembled a custom library of 70 kinase inhibitors (Supplemental Table S1) based on their targets and availability to identify compounds that can selectively affect distinct subsets of myeloid cells. Each inhibitor was added into the co-culture assay individually, and the cells were collected 4 days after co-culture and evaluated by flow cytometry. As shown above, the presence of TAb2 tumor cells promoted the expansion of TAMs (66.3%) in the group of BM+TAb2 (Figure 2A). Based on pilot experiments, we chose two concentrations to test our kinase inhibitors (100 nM and 10 μM) to survey their abilities to affect tumor-associated myeloid cells. We found that a group of inhibitors remarkably reduced the percentage of TAMs in the co-culture, especially when applied at 10 μM concentration (Figure 2A,B, Supplemental Figure S2). For instance, Pexidartinib significantly reduced the percentage of TAMs at both concentrations (17.0% for 100 nM, 3.20% for 10 μM), whereas Trametinib did so only at the higher concentration (51.1% for 100 nM vs. 2.08% for 10 μM) (Figure 2A,B).

**Figure 3:**
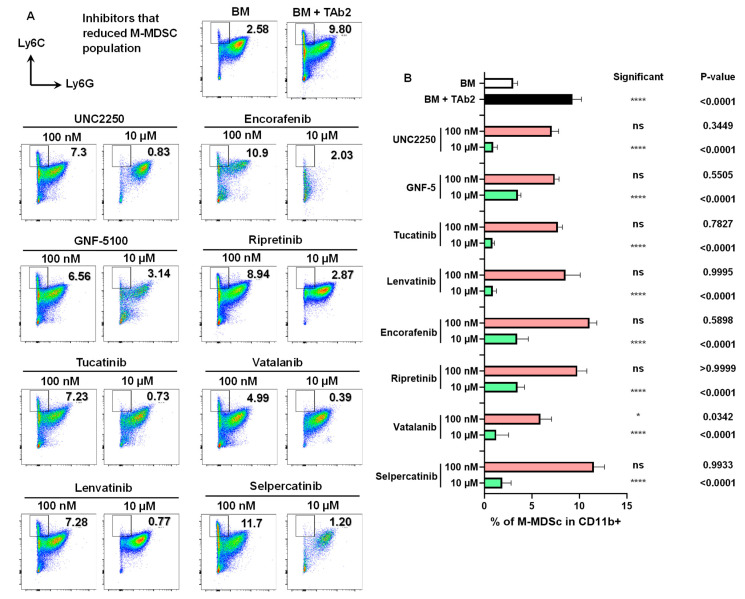
Inhibitors that reduced the M-MDSC Population. (**A**) Representative flow plots of M-MDSC (CD11b^+^Ly6C^high^Ly6G^−^) population in co-culture assay. Cells were treated and analyzed as described above in Figure 2. (**B**) Percentage of M-MDSC in the CD11b^+^ population. *p*-values of comparisons between BM (white bar) vs. BM+TAb2 (black bar) or between a given group (red or green bar) vs. BM+TAb2 group (black bar, positive control). Statistical significance was defined as * *p* < 0.05, **** *p* < 0.0001, ns: non-significant. Results are from three independent experiments performed in triplicate.

**Figure 4:**
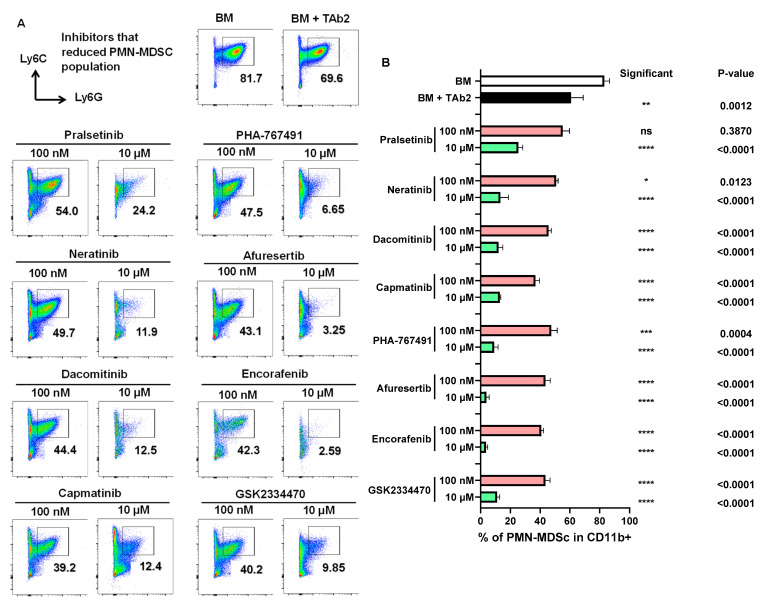
Inhibitors that reduced the PMN-MDSC population. (**A**) Representative flow plots of PMN-MDSCs (CD11b^+^Ly6C^low^Ly6G^+^) population in co-culture assay. Cells were treated and analyzed as described above in Figure 2. (**B**) Percentage of PMN-MDSCs in CD11b^+^ population. *p*-values of comparisons between BM (white bar) vs. BM+TAb2 (black bar) or between a given group (red or green bar) vs. BM+TAb2 group (black bar, positive control). Statistical significance was defined as * *p* < 0.05, ** *p* < 0.01, *** *p* < 0.001, **** *p* < 0.0001, ns: non-significant. Results are from three independent experiments performed in triplicate.

The presence of TAb2 tumor cells significantly promoted the expansion of M-MDSC compared with the BM-only control (Figure 3A,B, BM vs. BM+TAb2). We also identified inhibitors that reduced the M-MDSC population (Figure 3A,B). Notably, all identified inhibitors significantly reduced the percentage of M-MDSC at the higher concentration (10 μM) except vatalanib, which did so at both concentrations (100 nM and 10 μM) (Figure 3A,B). The presence of TAb2 tumor cells did not expand PMN-MDSC; instead, it significantly reduced the percentage of PMN-MDSC, compared with the BM-only control (Figure 4A), suggesting that a vast majority of BM cells remained as immature PMN-MDSC in the absence of other stimuli. We found specific inhibitors that reduced the percentage of PMN-MDSC (Figure 4A,B).

**Figure 5:**
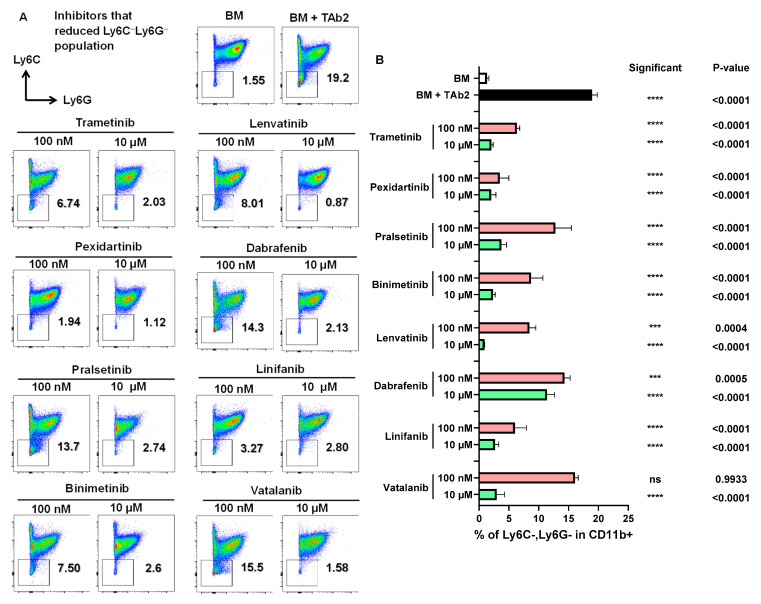
Inhibitors that reduced Ly6C^−^Ly6G^−^ double negative population. (**A**) Representative flow plots of Ly6C^−^Ly6G^−^ double negative population in co-culture assay. Cells were treated and analyzed as described above in Figure 2. (**B**) Percentage of Ly6C^−^Ly6G^−^ double negative population in CD11b^+^ population. *p*-values of comparisons between BM (white bar) vs. BM+TAb2 (black bar) or between a given group (red or green bar) vs. BM+TAb2 group (black bar, positive control). Statistical significance was defined as *** *p* < 0.001, **** *p* < 0.0001, ns: non-significant. Results are from three independent experiments performed in triplicate.

We noticed that the presence of TAb2 tumor cells drastically expanded the Ly6C^−^Ly6G^−^ population (Figure 5A,B, BM vs. BM+TAb2), and most of this consisted of F4/80^+^ macrophages (Figure 2A,B, BM+TAb2). We identified specific inhibitors that reduced the Ly6C^−^Ly6G^−^ population (Figure 5A,B), most of which (7 out of 8) overlapped with the inhibitors reducing the TAM population (CD11b^+^Ly6C^−^Ly6G^−^F4/80^+^) (Figure 2A,B), except Linifanib. Many other inhibitors also reduced the Ly6C^−^Ly6G^−^ population to a lesser extent (Supplemental Figure S3). Notably, we also uncovered a group of inhibitors that increased the percentage of the Ly6C^−^Ly6G^−^ population (gated on CD11b^+^) (Supplemental Figures S4A,B and S5A,C). Out of these inhibitors, some reduced the percentage of TAMs (Supplemental Figure S4C,D), whereas others did not affect it substantially (Supplemental Figure S5B,D). Additionally, we identified a group of inhibitors that do not have a significant effect on any of the populations (Supplemental Table S3). These results highlight the complexity of inhibitors’ effects on tumor-associated myeloid populations and caution their application as anti-cancer drugs. Taken together, we conclude that specific inhibitors affect the percentage of distinct subsets of tumor-associated myeloid cells.

### 2.3. In Vivo Validation of the Inhibitory Effects on Myeloid Cells by Vatalanib

**Figure 6:**
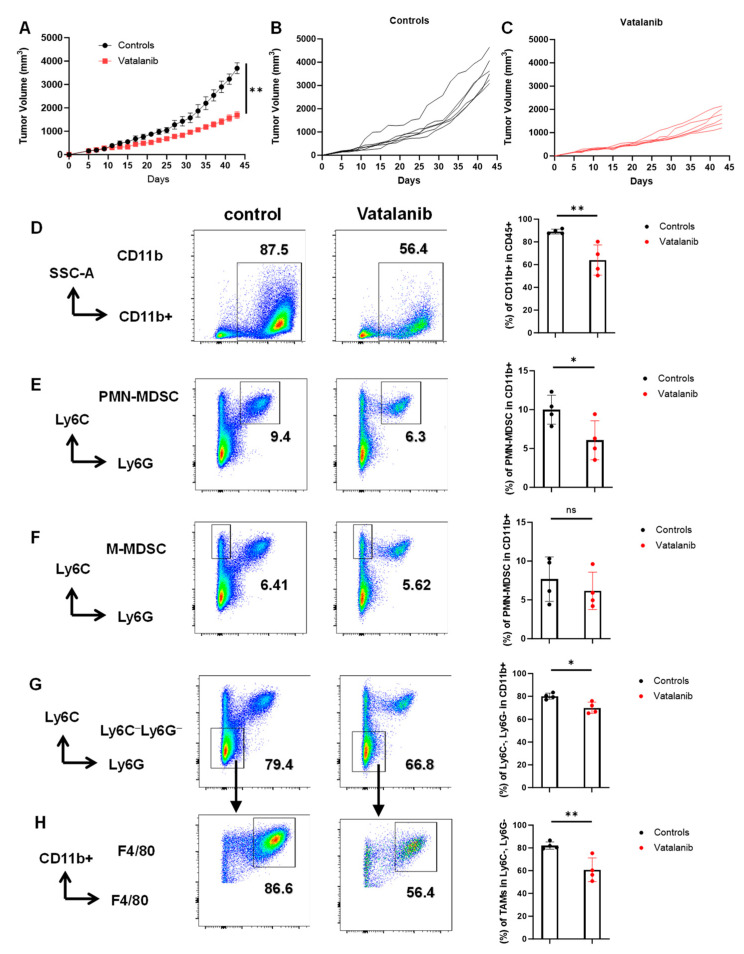
In vivo validation of the inhibitory effects of vatalanib on myeloid cells. (**A**–**C**) Tumor growth curves in the control or Vatalanib-treated tumor-bearing mice. TAb2 tumor cells were inoculated into WT B6 mice and randomized into two groups, treated with vehicle control (controls) (n = 6) or vatalanib (n = 6) (**A**). Individual tumor growth curves for controls (**B**) or vatalanib-treated ones (**C**). Tumor volume (TV, defined in Methods) was compared between the control and treated groups. (**D**–**H**). A flow cytometry analysis of tumor samples from tumor-bearing mice, treated with the control (n = 4) or vatalanib (n = 4), respectively, for all panels of (**D**–**H**). (**D**). Left: representative flow plots showing the CD11b^+^ population within the CD45^+^ population in the indicated groups. Right: quantification of the percentage of CD11b^+^ cells within the CD45^+^ population. (**E**) Left: representative flow plots of PMN-MDSCs (Ly6C^low^Ly6G^+^). Right: quantification of the percentage of PMN-MDSCs within the CD11b^+^ population. (**F**) Left: representative flow plots of M-MDSC (Ly6C^high^Ly6G^−^). Right: quantification of the percentage of M-MDSCs within the CD11b^+^ population. (**G**). Left: representative flow plots of the Ly6C^−^Ly6G^−^ double-negative population. Right: quantification of the percentage of the Ly6C^−^Ly6G^−^ double-negative population within the CD11b^+^ population. (**H**). Left: representative flow plots of TAMs gated on the Ly6C^−^Ly6G− population, displayed for CD11b^+^ vs. F4/80^+^. Right: quantification of the percentage of TAMs (CD11b^+^F4/80^+^Ly6C^−^Ly6G^−^) within the Ly6C^−^Ly6G^−^ population. The statistical significance was calculated using an unpaired *t*-test; * *p* < 0.05, ** *p* <  0.01, ns: non-significant.

We next chose vatalanib to validate our findings in the co-culture system because vatalanib reduced the percentage of TAMs, M-MDSC and Ly6C^−^Ly6G^−^ populations, while it did not affect the percentage of PMN-MDSC significantly in vitro (Supplemental Figure S6). TAb2 tumor cells were injected at the flank of WT B6 mice. When tumor size reached ~150–180 mm^3^, tumor-bearing mice were randomized into two groups and treated with vehicle control or vatalanib, respectively. We demonstrated that vatalanib treatment significantly reduced tumor growth (Figure 6A–C). Furthermore, we analyzed the tumor-infiltrating leukocytes by flow cytometry and showed that vatalanib significantly reduced the percentage of CD11b^+^ myeloid cells in CD45^+^ population (Figure 6D). It also reduced the percentage of PMN-MDSC and Ly6C^−^Ly6G^−^ population in CD11b^+^ population (Figure 6E,G), and the percentage of F4/80^+^ TAMs in the population of CD11b^+^Ly6C^−^Ly6G^−^ (Figure 6H). In summary, we validated the inhibitory effects of vatalanib on tumor-infiltrating myeloid cells in vivo.

## 3. Discussion

We employed a unique co-culture system to screen a custom library of 70 kinase inhibitors and identified specific inhibitors that reduced the expansion of different subsets of myeloid cells co-cultured with tumor cells. We also validated the effects of one inhibitor, vatalanib, on tumor growth and tumor-infiltrating myeloid cells using a in vivo tumor model. Our study not only highlights specific kinase inhibitors exhibiting differential effects on subsets of myeloid cells but also introduces a versatile experimental platform for identifying new agents targeting tumor-infiltrating myeloid cells in HNSCC. This platform can be readily adapted to test the effects of other types of cancer cells, such as melanoma, lung cancer, or breast cancer, on distinct subsets of myeloid cells. It is also possible to adapt this platform to other pre-clinical models, such as three-dimensional (3D) cultures or organoids, which may better mimic the in vivo TME [15,16]. Additionally, the number of inhibitors to be tested can be scaled up to accommodate large screens in a high throughput manner in order to identify novel and more effective targets.

We tested vatalanib due to its inhibitory effects on multiple subsets of myeloid cells. vatalanib inhibits all known VEGF receptors, platelet-derived growth factor receptor-β, and c-kit, while it is most selective for VEGFR-2 [17,18,19,20]. In this regard, we showed that Lenvatinib, which was approved for advanced renal cell carcinoma (RCC), also reduced the percentage of TAMs, M-MDSC, and the population of CD11b^+^Ly6G^−^Ly6C^−^ myeloid cells. Prior studies showed that lenvatinib plus pembrolizumab was associated with significantly longer progression-free survival and overall survival than sunitinib in advanced RCC patients [21], or than chemotherapy in patients with advanced endometrial cancer [22]. However, the combination of lenvatinib plus pembrolizumab did not lead to more benefits in patients with untreated metastatic NSCLC than pembrolizumab alone [23], and its effects on HNSCC remain unclear [24]. The effect of combined vatalanib and immune checkpoint inhibitor remains unknown in the context of cancer immunotherapy.

Notably, we discovered inhibitors that selectively increase the population of CD11b^+^Ly6G^−^Ly6C^−^ myeloid cells; moreover, some of them reduced TAM population while others did not (Supplemental Figures S4 and S5), adding another layer of complexity to the regulatory mechanisms governing myeloid cell dynamics. M-MDSC and PMN-MDSC subsets are relatively established and well-studied; however, the population of CD11b^+^Ly6G^−^Ly6C^−^ myeloid cells is less well characterized. Consistent with prior studies [14], we showed that most of CD11b^+^Ly6G^−^Ly6C^−^ population consisted of F4/80^+^ macrophages. However, the function of rest CD11b^+^Ly6G^−^Ly6C^−^ population (F4/80^−^) remain less well-known. A recent study suggested that depleting the CD11b^+^Ly6G^−^Ly6C^−^ population in brain tumor models enhanced survival and therapy responses [25]. While it is well-established that PMN-MDSCs have immunosuppressive functions [1,26], our recent study showed that PMN-MDSCs can be permissive of anti-tumor immunity under certain circumstances [27], consistent with a report showing that PMN-MDSC in ceralasertib, an ATR inhibitor, treated tumor-bearing mice were less suppressive against CD8 T cells, which was associated with up-regulation of type I IFN signature [28].

While our studies focus on the effects of inhibitors on myeloid cells, it is conceivable that the phenotypes or functions of tumor cells are also affected in the co-culture system in the presence of specific inhibitors. It may be worthwhile to investigate tumor cell-intrinsic changes using our unique system that provides an opportunity to dissect how tumor and myeloid cells influence each other. Advancing the understanding of tumor-myeloid cell interaction may pave the way for developing targeted therapies aimed at overcoming the immunosuppressive barriers in HNSCC and beyond.

Despite the promising findings of our study, several limitations should be acknowledged. (1) Our experiments were conducted using one cell line, which may limit the generalizability of our results. The response of tumor-associated myeloid cells to kinase inhibitors could vary significantly across different HNSCC cell lines or other cancer types. One of our primary goals was to provide an experimentally tractable system for the community to study the interplay between tumor cells and myeloid cells. We hope that researchers can benefit from our findings and consider applying these approaches to other types of cancers or to other cell lines of interest. (2) While our unique co-culture system allowed for insights into tumor-myeloid cell interactions, it may not fully replicate the complex TME found in vivo. Factors such as extracellular matrix composition, immune cell interactions, and systemic influences could alter the behavior of myeloid cells in a clinical setting. Other pre-clinical models may be useful in this context [15,16]. (3) Although our library comprised 70 kinase inhibitors, this selection may not encompass all potential therapeutic agents. There are numerous other targets that could also be explored to identify additional effective treatments for myeloid cells associated with HNSCC or beyond.

## 4. Materials and Methods

### 4.1. Cell Proliferation Assay

The proliferation of bone marrow (BM) cells was examined using the CellTrace™ Violet Cell Proliferation Kit (Cat. No., C34557 Thermo Fischer Scientific, Waltham, MA, USA) according to the manufacturer’s instruction. The BM tissue was isolated from wild-type (WT) C57BL/6 (B6) mice (JAX Lab, Bar Harbor, ME, USA) (6–8 weeks, both male and female) using a 25-G needle syringe (Thermo Fischer Scientific, Waltham, MA, USA), as described previously [14]. BM cells were filtered through a 70 μm cell strainer, and red blood cells (RBCs) were lysed using a ACK lysis buffer (Quality Biological, Gaithersburg, MD, USA). BM cells were resuspended in 5 μm CellTrace Violet in 1 × PBS, incubated at room temperature (RT) for 20 min in the dark, then neutralized with culture medium, followed by an additional 5 min incubation at RT in the dark. CellTrace Violet labeled cells were then washed with PBS, and BM cells (1 × 10^6^/well) were added into the culture plates (24-well) in triplicate either alone (BM only) or with TAb2 tumor cells (BM+TAb2) (Day 0). One day prior to the co-culture of BM and tumor cells, TAb2 tumor cells were resuspended in DMEM complete media [14] and plated in culture (2.5 × 10^4^/well in a 24-well plate) (Day −1). On Day 0, the culture supernatant of tumor cells was removed, and BM cells in RPMI complete media [29] were added into the culture plates. After 4 days of culture, cells were harvested, stained with the LIVE/DEAD™ Fixable Green Dead Cell Stain Kit (Catalog No. L23101, Invitrogen, Waltham, MA, USA), and analyzed for CellTrace Violet intensity using flow cytometry. Flow acquisition was performed on a BD Fortessa (BD Biosciences, Milpitas, CA, USA). Data were processed and analyzed using FlowJo™ software version 10.9.0 (FLOWJO, Ashland, OR, USA).

### 4.2. Cell Culture and Inhibitor Treatment

The parental TAb2 cell line was derived from a spontaneous tumor that developed in K15.CrePR1(+)p53f/fPIK3CAc/c mouse [30]. Then, the parental TAb2 tumor line was transplanted into wild-type (WT) C57BL/6 (B6) recipients (Jax Laboratories, Bar Harbor, ME, USA) [14]. Transplanted tumors were isolated and used for histology analysis and for creating a daughter TAb2 cell line [14] that was employed for the current study. TAb2 tumors are poorly differentiated SCC expressing cytokeratin 5 (CK5), a marker for SCCs, and vimentin (Vim), a mesenchymal marker [14]. TAb2 tumor cells were cultured in DMEM complete media supplemented with a 10% fetal bovine serum (FBS), 1% penicillin/streptomycin, and a 1% HEPES buffer at 37 °C CO_2_ incubator (5%) [14]. We selected TAb2 tumor cells to perform the current study due to the following reasons: (1) this tumor cell line generated a TME highly enriched with tumor-infiltrating myeloid cells in vivo [14]; (2) we have employed this tumor cell line in previous studies and showed that it caused the expansion of TAM [14] demonstrating the feasibility of using this tumor cell line, although the mechanisms of such an observation were not investigated in a prior study [14]; (3) given the proven feasibility of Tab2 tumor cells in the co-culture system, we aim to identify specific inhibitors that could affect phenotypes of tumor-associated myeloid cells, ultimately leading to the discovery of new therapeutic targets. The inhibitors were purchased from Selleck Chemicals (Houston, TX, USA) (see Supplementary Table S1 for a customized small library of 70 inhibitors) and dissolved in DMSO, according to the manufacturer’s instructions. Each inhibitor was added into the suspension of BM cells at two concentrations, 100 nM or 10 μM, and the treated BM cells, together with the inhibitor, were added into the culture plates containing tumor cells on Day 0. BM control cells were cultured in RPMI complete media until day 4. RPMI complete media contained RPMI1640 medium supplemented with 10% fetal bovine serum (FBS), 100μM of β2-mercaptoethanol (M3148-100ML, Sigma, Burlington, MA, USA), 1% of antibiotics (15240-062, Gibco, Waltham, MA, USA), L-glutamine (25030-081, Gibco), MEM non-essential amino acid (25-025-CI, Corning, Gilbert, AZ, USA), HEPES (25-060-CI, Corning) and sodium pyruvate (11360-070, Gibco). On day 4, cultured cells were harvested and analyzed using the LIVE/DEAD™ Fixable Aqua Dead Cell Stain (Catalog No. L34966, Invitrogen, Waltham, MA, USA), and subsequently stained with flow antibodies (Ab) for the myeloid populations (see Ab in Supplementary Table S2). Flow cytometry was performed as described above.

### 4.3. In Vivo Animal Studies and Tumor Injection

WT B6 mice were purchased from Charles River (6–8 weeks). All mice were maintained under specific pathogen-free conditions in the UPMC Hillman Cancer Center Animal Facility (Pittsburgh, PA, USA). The animal work was approved by the Institutional Animal Care and Use Committee (IACUC) of the University of Pittsburgh.

TAb2 tumor cells were cultured and trypsinized as described previously [31]. The cells were resuspended in sterile PBS and Matrigel (Corning, AZ, USA) in a 1:1 ratio (50% PBS, 50% Matrigel). Subsequently, 0.5 × 10^6^ tumor cells (in 100 μL volume) were injected subcutaneously at the flank regions of each mouse. When the tumor volume reached ~150–180 mm^3^, tumor-bearing mice were randomized into two groups and treated with vehicle control or vatalanib (Med Chem Express, Monmouth Junction, NJ, USA, Catalog# HY-12018/CS-0149) diluted in polyethylene glycol 400 (PEG400) via oral gavage at a dose of 50 mg/kg [17]. Treatment was administered in seven doses, every other day, starting from day 7 post tumor injection. PEG400 was used as the vehicle control (VC). Tumor length and width were measured with calipers, and tumor volume (TV) was calculated as (length × width^2^)/2.

### 4.4. Analysis of Tumor-Infiltrating Myeloid Cells

Single-cell suspensions were prepared from tumors harvested from tumor-bearing mice, as previously described [30]. Briefly, the tumors were finely chopped and digested with Liberase DL (50 μg/mL) (Roche, Atlanta, GA, USA) at 37 °C for 30 min. After digestion, samples were filtered through 70 μm cell strainers. RBCs in the suspension were lysed using ACK lysis buffer (Quality Biological, Gaithersburg, MD, USA) and the cell suspension was then neutralized with the medium. Cells were washed and centrifuged at 1500 rpm for 5 min at 4 °C. The resulting single-cell suspension was first stained with LIVE/DEAD Fixable Aqua Dead Cell Stain (Invitrogen, Waltham, MA, USA), then blocked with TruStain FcX CD16/32 (BioLegend, San Diego, CA, USA) following the manufacturer’s instructions, and stained with Abs for myeloid cells used at the recommended concentrations (See Ab in Supplementary Table S2). Flow cytometry analysis was performed as described above.

## Data Availability

All data are included in the manuscript and Supplemental Information.

## Supplementary Materials

The following supporting information can be downloaded at: https://www.mdpi.com/article/10.3390/ijms252212277/s1.

## Abbreviations

HNSCCs: Head and Neck Squamous Cell Carcinomas; HPV: Human Papillomavirus; Ab: Antibody; WT: Wild-Type; B6: C57BL/6; BM: Bone-Marrow; RBC: Red Blood Cells; MDSCs: Myeloid-Derived Suppressor Cells; M-MDSC: Monocytic MDSC; PMN-MDSC: Polymorphonuclear MDSC; TAMs: Tumor-Associated Macrophages; TME: Tumor Microenvironment; TV: Tumor Volume; VEGF: Vascular Endothelial Growth Factor; VEGFR: Vascular Endothelial Growth Factor Receptor; VC: Vehicle Control.
