# Supplementary material for: Targeting Myeloid Cells in Head and Neck Squamous Cell Carcinoma: A Kinase Inhibitor Library Screening Approach
Source: Int J Mol Sci. 2024 Nov 15;25(22):12277. doi: 10.3390/ijms252212277 (PMC11595410; doi:10.3390/ijms252212277)
Supplement: Supplementary file 1 [file ijms-25-12277-s001.zip › Suplemantray Figures-11-6-2024.pdf]

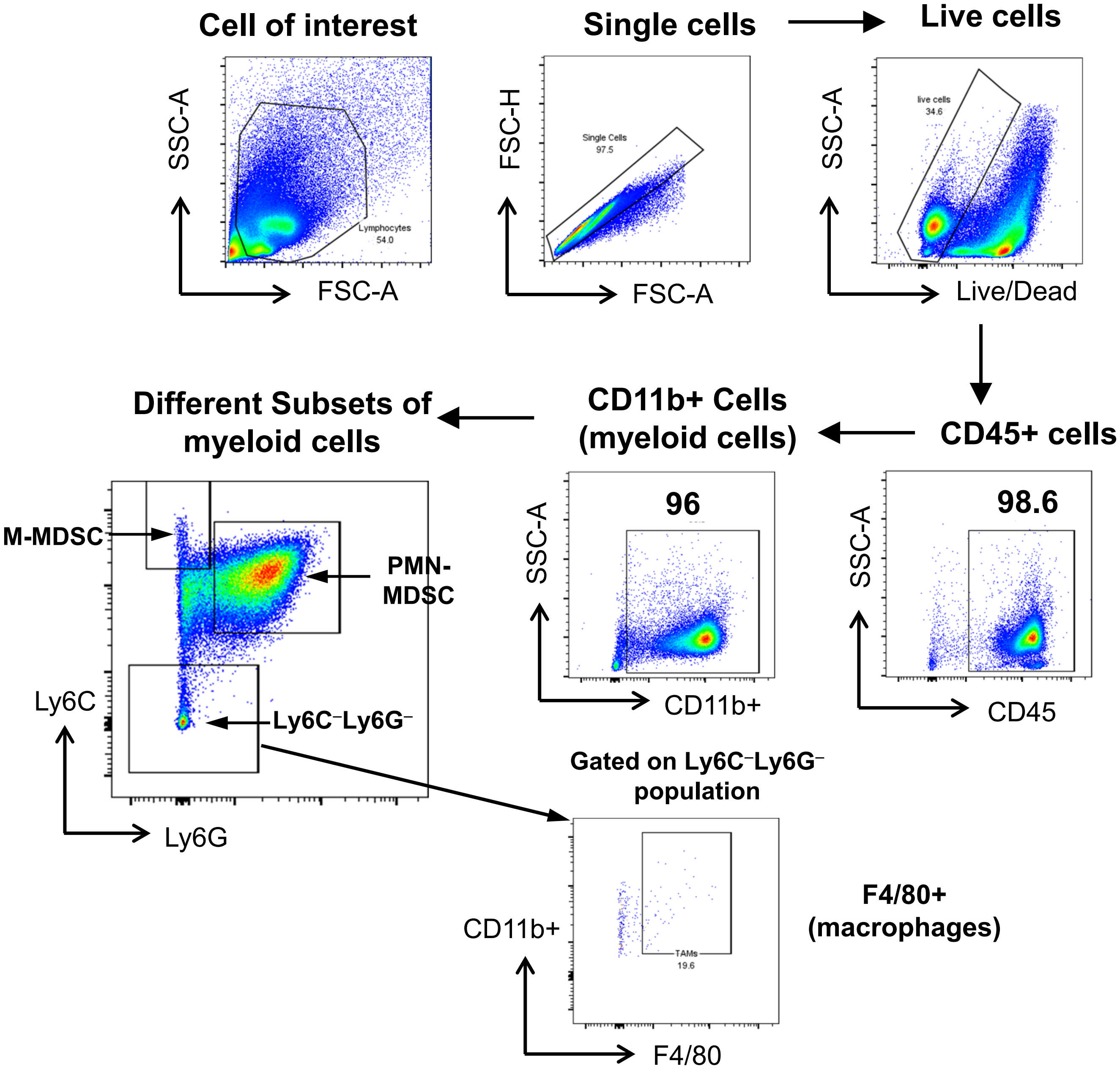


**Supplemental Figure S1. Gating strategy for subsets of myeloid cells in BM cells cultured alone.** Representative flow plots from BM cells cultured alone after 4 days. After gating on the $CD45^{+}$ population, $CD11b^{+}$ population was gated for myeloid cells. Within the $CD11b^{+}$ population, M-MDSCs ($Ly6C^{high}Ly6G^{-}$), PMN-MDSCs ($Ly6C^{low}Ly6G^{+}$), and double-negative ($Ly6C^{-}Ly6G^{-}$) populations were shown. After gating on $Ly6C^{-}Ly6G^{-}$ population, TAMs were shown ($F4/80^{+}CD11b^{+}$).

Supplemental Figure S1

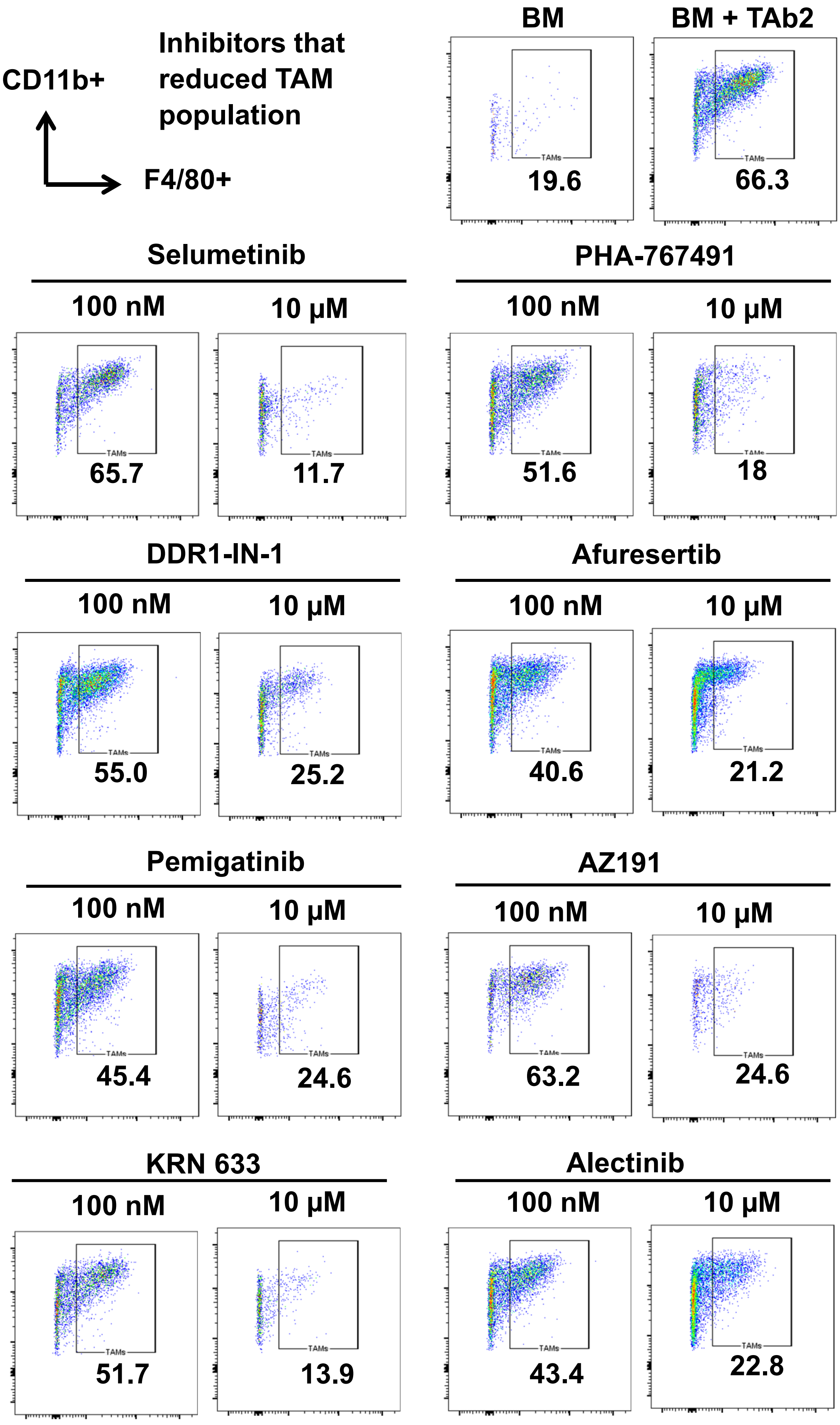


Supplemental Figure S2A

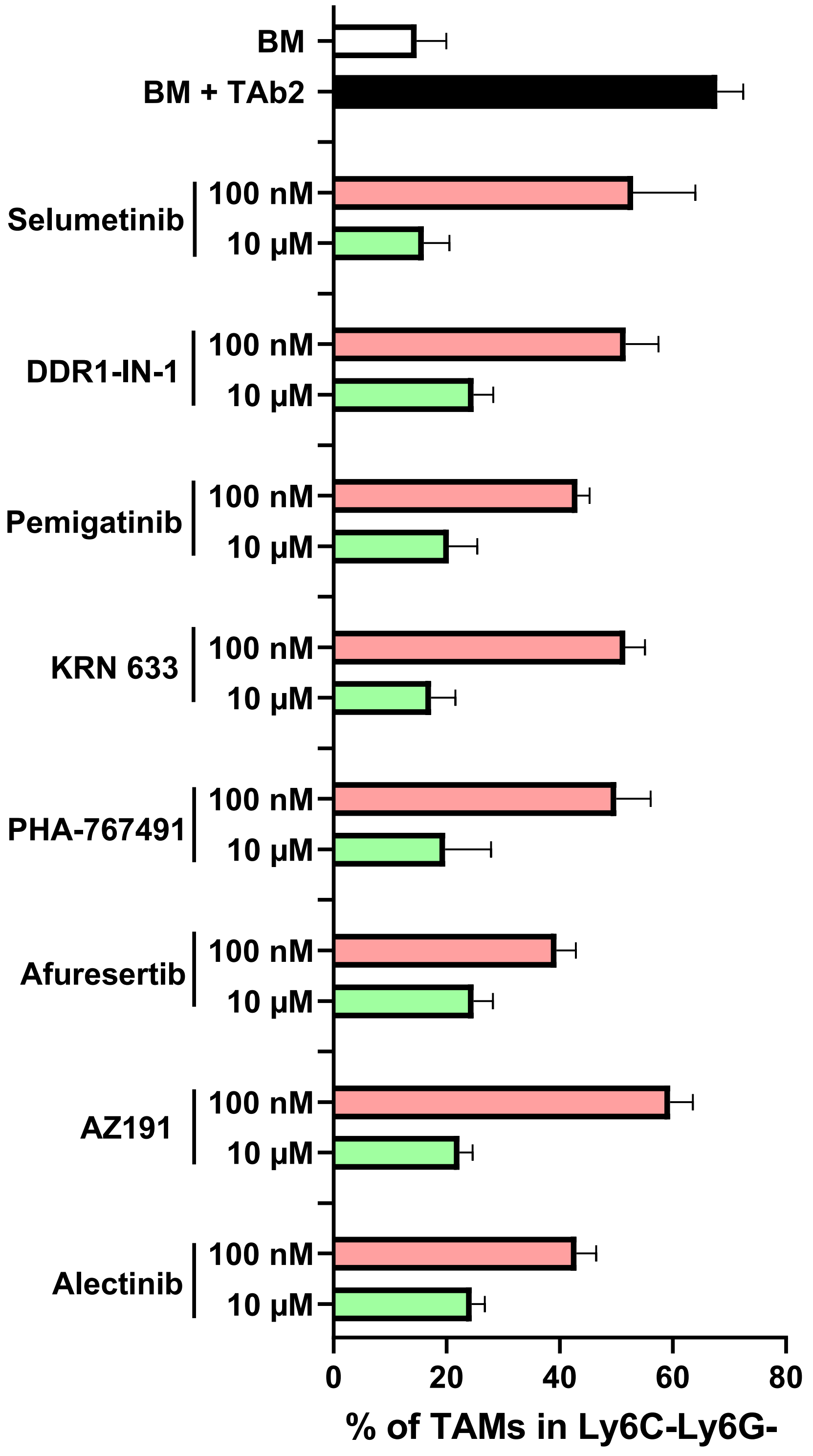


| Significant | P-value |
|---|---|
| *** | <0.0001 |
| * | 0.0124 |
| **** | <0.0001 |
| ** | 0.0053 |
| **** | <0.0001 |
| **** | <0.0001 |
| **** | <0.0001 |
| ** | 0.0051 |
| **** | <0.0001 |
| ** | 0.0017 |
| **** | <0.0001 |
| **** | <0.0001 |
| **** | <0.0001 |
| ns | 0.3803 |
| **** | <0.0001 |
| **** | <0.0001 |
| **** | <0.0001 |

Supplemental Figure S2B

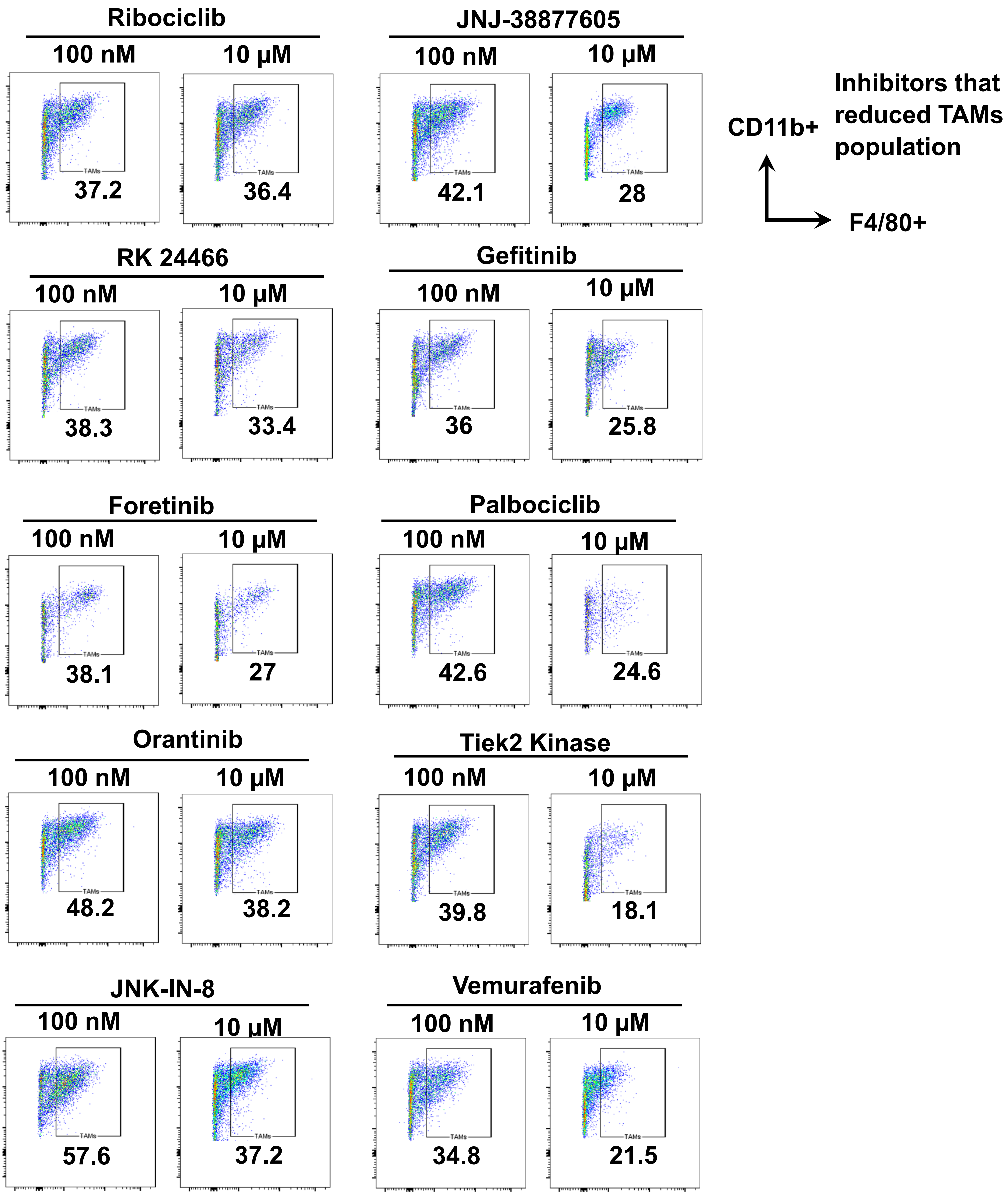


Supplemental Figure S2C

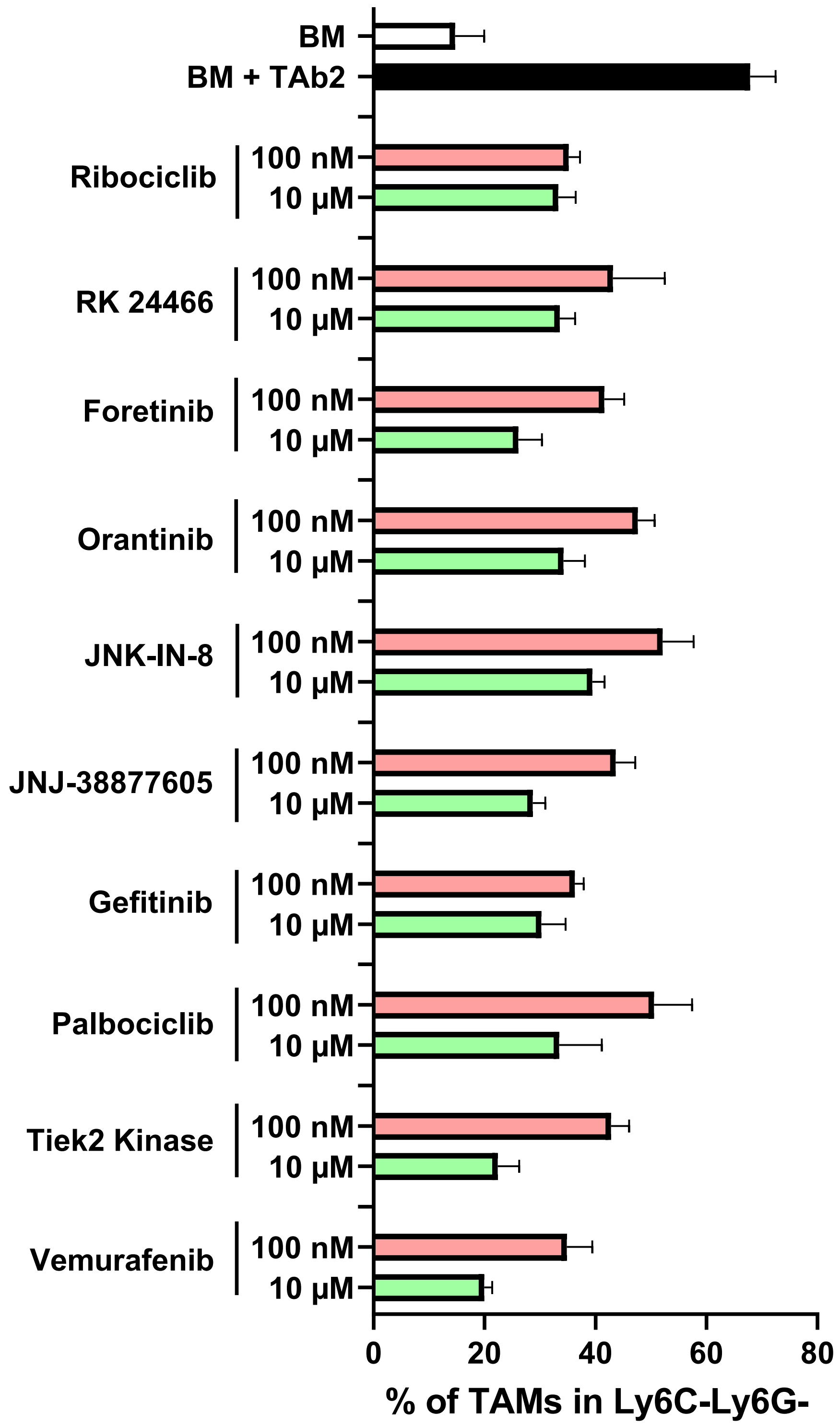


| Significant | P-value |
|---|---|
| **** | <0.0001 |
| **** | <0.0001 |
| **** | <0.0001 |
| **** | <0.0001 |
| **** | <0.0001 |
| **** | <0.0001 |
| **** | <0.0001 |
| **** | <0.0001 |
| **** | <0.0001 |
| **** | <0.0001 |
| **** | <0.0001 |
| ** | 0.0016 |
| **** | <0.0001 |
| **** | <0.0001 |
| **** | <0.0001 |
| **** | <0.0001 |
| **** | <0.0001 |
| *** | 0.0004 |
| **** | <0.0001 |
| **** | <0.0001 |
| **** | <0.0001 |

Supplemental Figure S2D

**Supplemental Figure S2. Inhibitors that reduced TAMs Populations**. **A,C** Representative flow plots of CD11b$^+$F4/80$^+$ TAMs in co-culture assays. BM and BM+TAb2 serve as the negative (19.6%) and positive (66.3%) control, respectively. Indicated kinase inhibitors (100nm or 10$\mu$M) were added into co-culture. Cells were analyzed 4 days after culture. TAMs: CD11b$^+$Ly6C$^-$Ly6G$^-$F4/80$^+$. **B,D** Percentage of TAMs in Ly6C$^-$Ly6G$^-$ population. *P* values of comparisons between BM (white bar) vs. BM+TAb2 (black bar) or between a given group (red or green bar) vs. BM+TAb2 group (black bar, positive control) were determined using one-way ANOVA with Tukey's multiple comparison test. Statistical significance was defined as *p<0.05, **p< 0.01, ***p<0.001, ****p<0.0001. Results are from three independent experiments performed in triplicates.

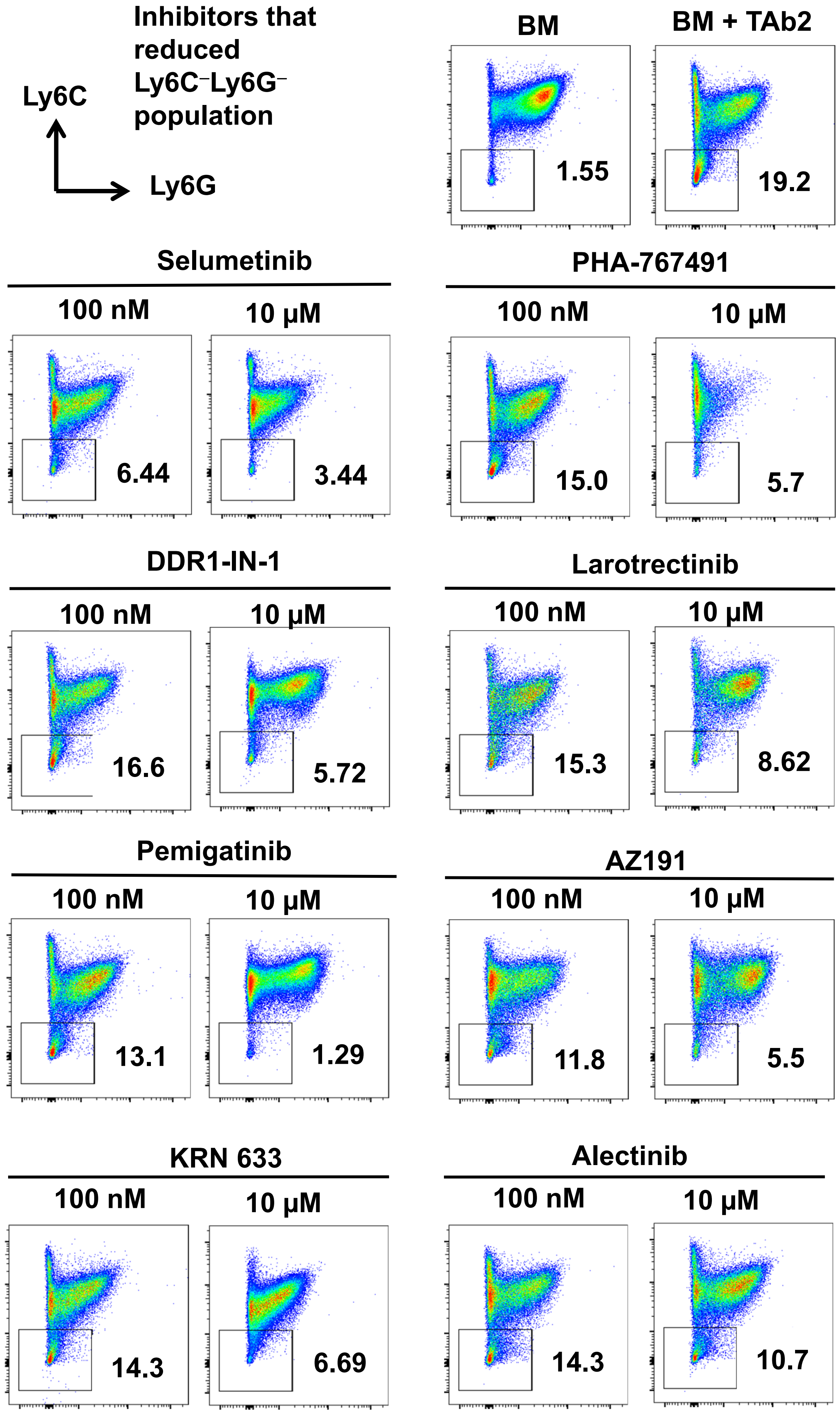


Supplemental Figure S3A

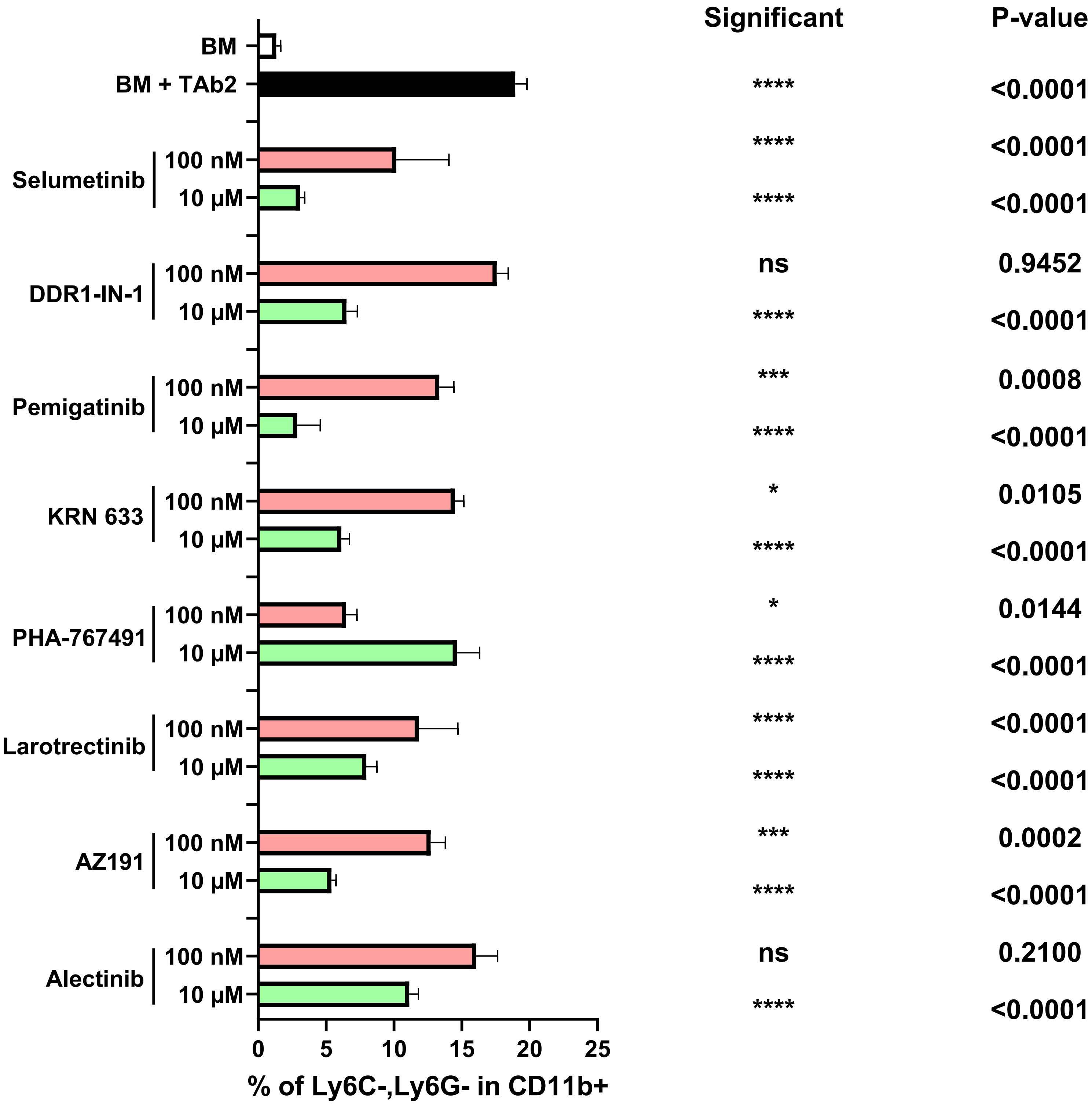


Supplemental Figure S3B

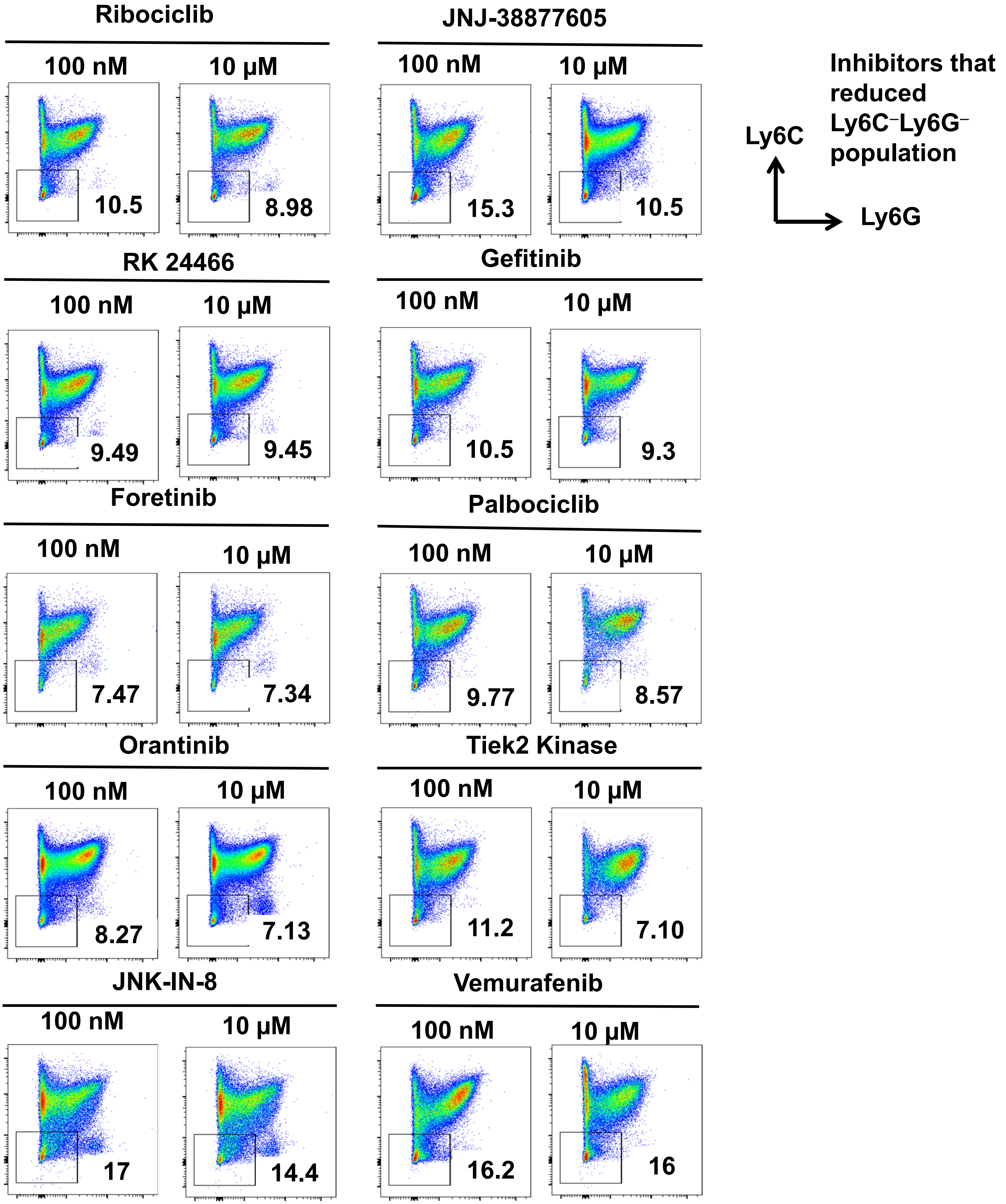


Supplemental Figure S3C

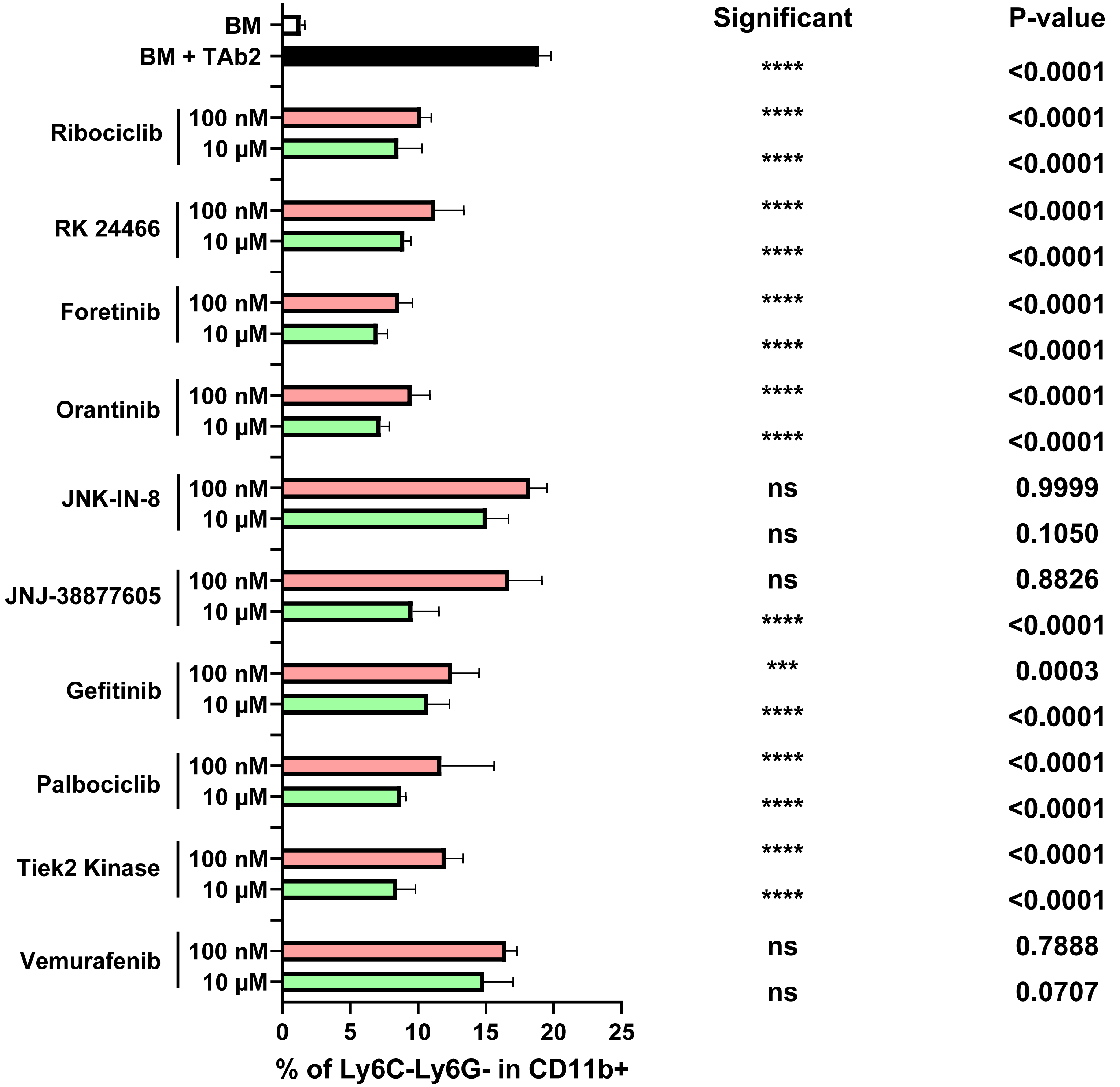


Supplemental Figure S3D

**Supplemental Figure S3. Inhibitors that reduced Ly6C$^{-}$Ly6G$^{-}$ double negative population. A,C** Representative flow plots of Ly6C$^{-}$Ly6G$^{-}$ double negative population in co-culture assays. BM and BM+TAb2 serve as the negative (1.55%) and positive (19.2%) control, respectively. Indicated kinase inhibitors (100nm or 10μM) were added into co-culture. Cells were analyzed 4 days after culture. Gated population: CD11b$^{+}$Ly6C$^{-}$Ly6G$^{-}$. **B,D** Percentage of Ly6C$^{-}$Ly6G$^{-}$ population in CD11b$^{+}$ population. *P* values of comparisons between BM (white bar) vs. BM+TAb2 (black bar) or between a given group (red or green bar) vs. BM+TAb2 group (black bar, positive control) were determined using one-way ANOVA with Tukey's multiple comparison test. Statistical significance was defined as *$p<0.05$, ***$p<0.001$, ****$p<0.0001$. Results are from three independent experiments performed in triplicates.

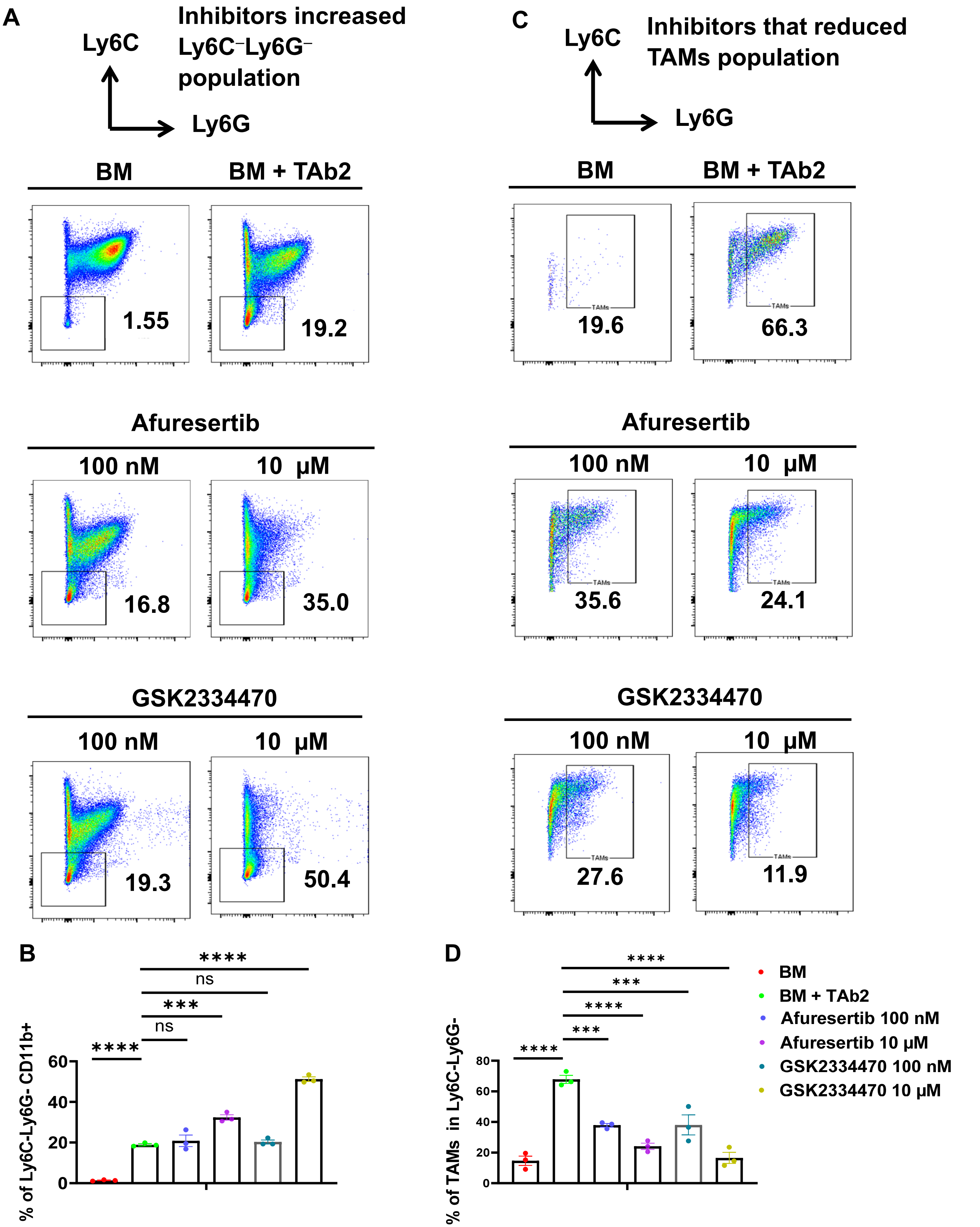


Supplemental Figure S4

**Supplemental Figure S4. Inhibitors that increased Ly6C$^{-}$Ly6G$^{-}$ double negative population but reduced TAMs.** BM and BM+TAb2 serve as the negative (1.55%) and positive (19.2%) control, respectively. Indicated kinase inhibitors (100nm or 10μM) were added into co-culture. Cells were analyzed 4 days after culture. **A,** Representative flow plots of Ly6C$^{-}$Ly6G$^{-}$ double negative population in co-culture assays. Gated population: CD11b$^{+}$Ly6C$^{-}$Ly6G$^{-}$. **B,** Quantification of the percentage of Ly6C$^{-}$Ly6G$^{-}$ double negative population in CD11b$^{+}$ population. **C,** Representative flow plots of TAMs in Ly6C$^{-}$Ly6G$^{-}$ population. **D,** Quantification of the percentage of TAMs in Ly6C$^{-}$Ly6G$^{-}$ population. *P* values of comparisons between BM (white bar) vs. BM+TAb2 (black bar) or between a given group (red or green bar) vs. BM+TAb2 group (black bar, positive control) were determined using one-way ANOVA with Tukey's multiple comparison test. Statistical significance was defined as ***p<0.001, ****p<0.0001. Results are from three independent experiments performed in triplicates.

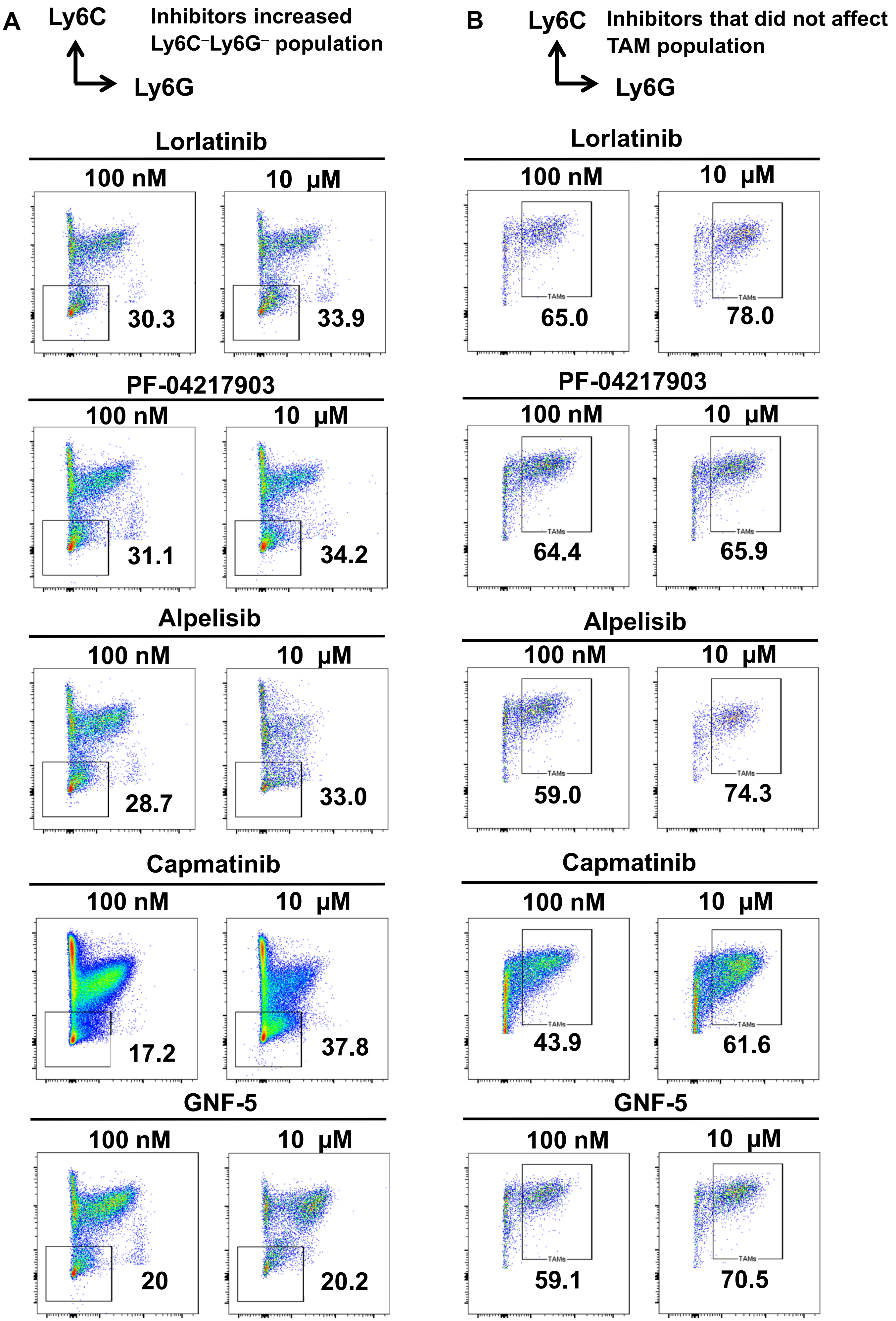


Supplemental Figure S5A

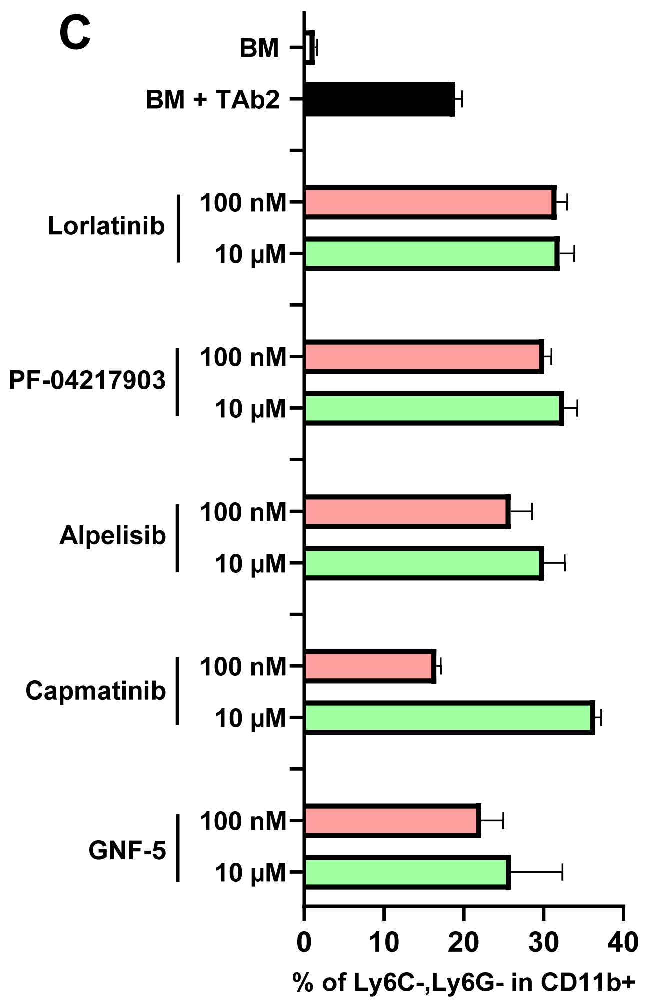


| Significant | P-value |
|---|---|
| **** | <0.0001 |
| **** | <0.0001 |
| **** | <0.0001 |
| *** | 0.0002 |
| **** | <0.0001 |
| * | 0.0222 |
| *** | 0.0002 |
| ns | 0.8586 |
| **** | <0.0001 |
| ns | 0.5952 |
| * | 0.0214 |

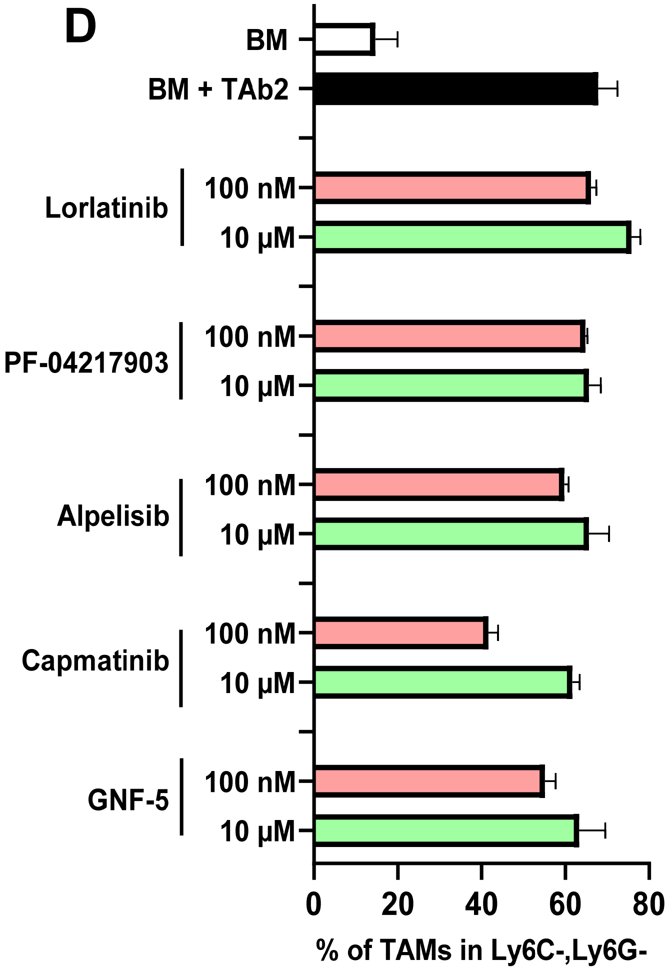


| Significant | P-value |
|---|---|
| **** | <0.0001 |
| ns | 0.9998 |
| ns | 0.0977 |
| ns | 0.9751 |
| ns | 0.9983 |
| ns | 0.0879 |
| ns | 0.9983 |
| **** | <0.0001 |
| ns | 0.3457 |
| ** | 0.0015 |
| ns | 0.7540 |

**Supplemental Figure S5B**

**Supplemental Figure S5. Inhibitors that increased Ly6C⁻Ly6G⁻ double negative population but did not affect TAMs substantially.** Indicated kinase inhibitors (100nm or 10μM) were added into co-culture of BM and TAb2 tumor cells. Cells were analyzed 4 days after culture. **A,** Representative flow plots of $Ly6C^{-}Ly6G^{-}$ double negative population in co-culture assays. Gated population: $CD11b^{+}Ly6C^{-}Ly6G^{-}$. **B,** Representative flow plots of TAMs in gated $Ly6C^{-}Ly6G^{-}$ population. **C,** Quantification of the percentage of $Ly6C^{-}Ly6G^{-}$ double negative population in $CD11b^{+}$ population. **D,** Quantification of the percentage of TAMs in $Ly6C^{-}Ly6G^{-}$ population. *P* values of comparisons between BM (white bar) vs. BM+TAb2 (black bar) or between a given group (red or green bar) vs. BM+TAb2 group (black bar, positive control) were determined using one-way ANOVA with Tukey's multiple comparison test. Statistical significance was defined as *p<0.05, **p<0.01, ***p<0.001, ****p<0.0001. Results are from three independent experiments performed in triplicates.

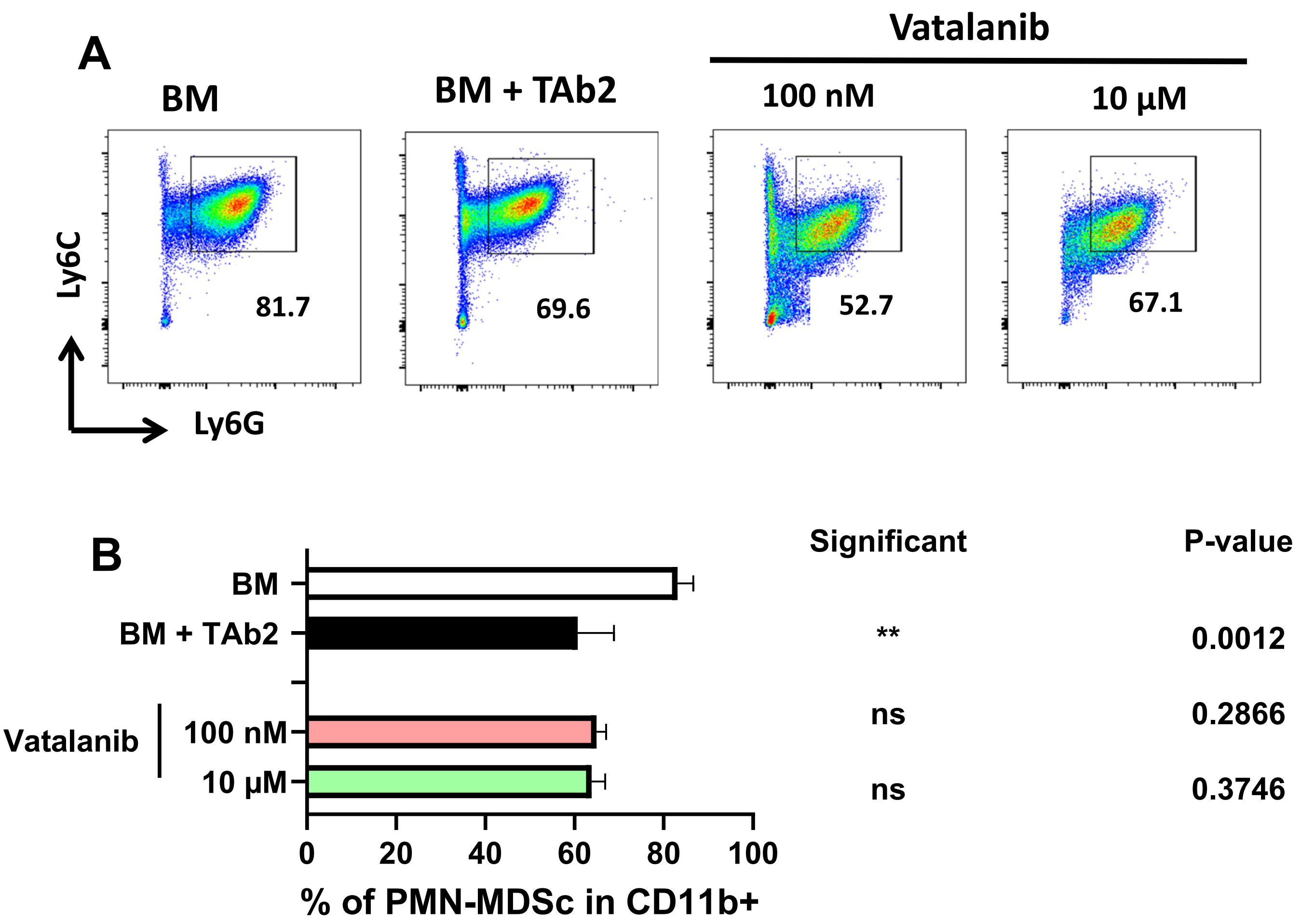


**Supplemental Figure S6. Effect of Vatalanib on PMN-MDSC Population in vitro.** **A,** Representative flow plots of PMN-MDSCs ($CD11b^{+}Ly6C^{low}Ly6G^{+}$) population in co-culture assay. Cells were treated and analyzed as described above in Figure 2. **B,** Percentage of PMN-MDSCs in $CD11b^{+}$ population. *P* values of comparisons between BM (white bar) vs. BM+TAb2 (black bar) or between a given group (red or green bar) vs. BM+TAb2 group (black bar, positive control) were determined using one-way ANOVA with Tukey's multiple comparison test. Results are from three independent experiments performed in triplicate.

**Supplemental Figure S6A, B**
