# Supplementary material for: Targeting Myeloid Cells in Head and Neck Squamous Cell Carcinoma: A Kinase Inhibitor Library Screening Approach
Source: Int J Mol Sci. 2024 Nov 15;25(22):12277. doi: 10.3390/ijms252212277 (PMC11595410; doi:10.3390/ijms252212277)
Supplement: Supplementary file 1 [file ijms-25-12277-s001.zip › Supplemental Table S2 FACS Antibody.pdf]

Supplementary Table S2. FACS Antibodies used in the study.

FACS Antibody	Fluorophore	Catalog	Clone	Company
CD45	BUV395	564279	30-F11	BD Bioscience
CD11b	FITC	101205	M1/70	BioLegend
Ly6G	APC/Cy7	127623	1A8	BioLegend
Ly6C	BV 421	128031	HK1.4	Biolegend
F4/80	PE Dazzle	123145	BM8	Biolegend
